# Breaking the SHG-stability trade-off in UV nonlinear optical materials by polar-layer-dense-locking

**DOI:** 10.1039/d6sc03803d

**Published:** 2026-05-29

**Authors:** Jiaqi Qian, Yunjie Wang, Jiafu Ding, Xin Su, Qi Wu

**Affiliations:** a State Key Laboratory of New Textile Materials and Advanced Processing, Wuhan Textile University Wuhan 430200 China wuqi2011@whu.edu.cn; b Xinjiang Laboratory of Phase Transitions and Microstructures in Condensed Matter Physics, College of Physical Science and Technology, Yili-Normal University Xinjiang 835000 China

## Abstract

In ultraviolet (UV) nonlinear optical (NLO) metal–organic complexes (MOCs), simultaneously achieving a strong second-harmonic generation (SHG) response and high thermal and water stability has remained a persistent bottleneck. Herein, a “polar-layer-dense-locking” strategy is utilized to synthesize a 3D cadmium-based coordination polymer, Cd(imc) (imc^2−^ = imidazole-4-carboxylate), breaking the long-standing SHG-stability trade-off. Employing the rigid, π-conjugated imc^2−^ ligand with large molecular hyperpolarizability (|*β*_max_|) as the “nonlinear source”, this strategy constructs high-performance 2D layers *via* µ_2_-κ^2^-N, O dual-bridging coordination. This architecture enforces a cooperative alignment of ligand dipoles, efficiently translating microscopic |*β*_max_| into an amplified macroscopic SHG response. The 3D topological network is achieved *via* strong Cd–O coordination bonds and dense interlayer packing, imparting exceptional structural stability. Cd(imc) exhibits a giant SHG response (10.0 × KDP), ultrahigh thermal stability (up to 470 °C), and remarkable water resistance (retains crystallinity for >180 days in water at room temperature and >30 days at 100 °C), alongside a wide bandgap (4.28 eV). It also exhibits good phase-matchability, making it a practical candidate for UV frequency conversion. This “polar-layer-dense-locking” paradigm establishes a synergistic mechanism to decouple the SHG-stability constraints in UV NLO materials.

## Abstract 2

The “polar-layer-dense-locking” strategy affords 3D Cd(imc) with ordered polar stacking. The material has SHG of 10.0 × KDP, stability to 470 °C, and stable water resistance (180 d at RT, 30 d at 100 °C).

## Graphical abstract

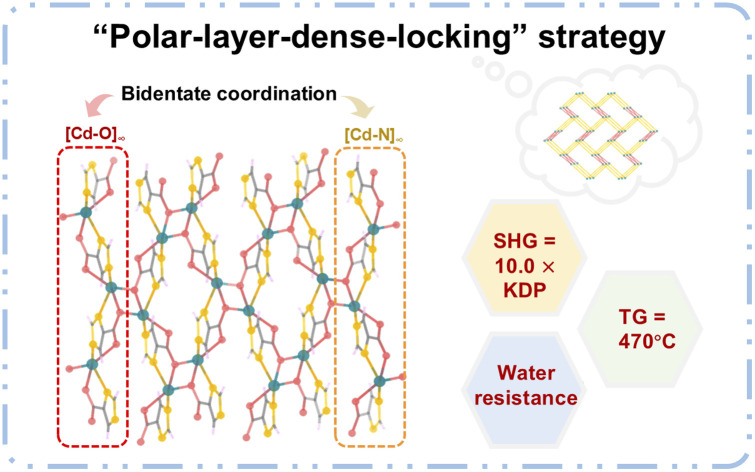

## Introduction

**Fig. 1:**
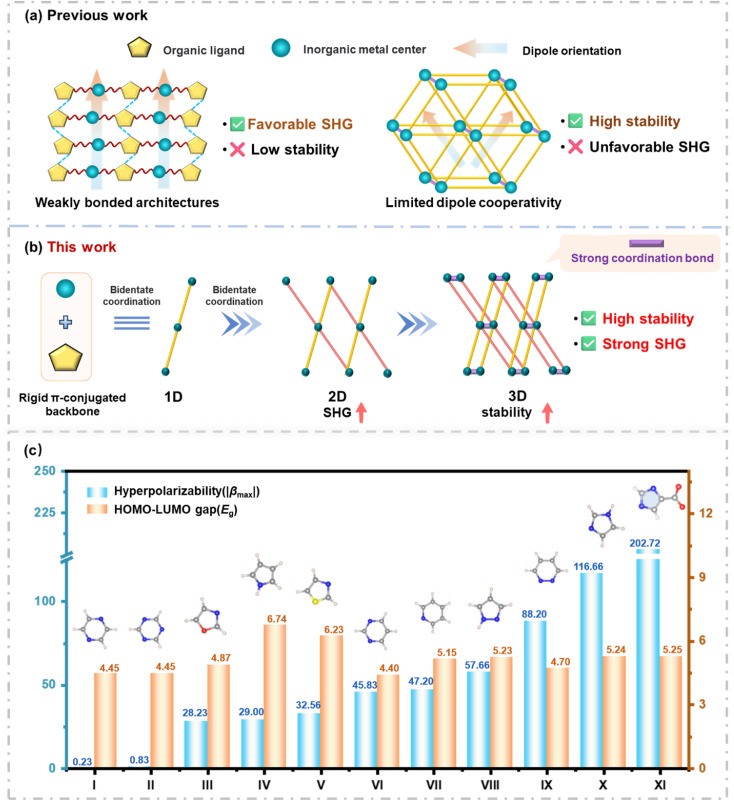
(a) Previous work. (b) 3D structure designed based on a crystal-engineering strategy utilizing the “polar-layer-dense-locking” synergistic mechanism. (c) Hyperpolarizability (|*β*_max_|) and HOMO–LUMO gap (*E*_g_) of N-containing rigid planar π-conjugated groups.

Ultraviolet (UV) nonlinear optical (NLO) crystals facilitate laser frequency conversion through second-harmonic generation (SHG) and serve as key components in high-power laser processing, optoelectronic devices, and quantum optics experiments.^1–5^ Metal–organic complexes (MOCs), combining the polarizability of metal centers with the structural tunability of organic ligands, are considered an ideal platform for constructing novel UV NLO crystals and have emerged as an important branch of NLO materials.^6–9^ High-performance UV NLO materials are required to simultaneously exhibit a large SHG coefficient, a wide optical bandgap, and sufficient phase-matching capability.^10–13^ In addition to the aforementioned challenges, UV NLO MOCs have long been plagued by a persistent trade-off between potent nonlinear optical activity and robust structural stability (Fig. 1a).

On one hand, highly polarizable or π-conjugated ligands enhance molecular hyperpolarizability (|*β*_max_|), and flexible linkers or weak hydrogen bonds help align the molecular dipoles, thereby enhancing the crystal SHG response, but at the same time limit framework densification and reduce structural stability.^14–18^ For example, Cd(SCN)_2_(C_4_H_6_N_2_)_2_ features directionally aligned, distorted [CdN_4_S] polar units that underpin a strong SHG response of 10.0 × KDP; however, the framework is primarily stabilized by hydrogen bonds and the organic ligands are prone to thermal decomposition, resulting in a decomposition temperature of 180 °C;^19^ (C_3_N_6_H_7_)(C_3_N_6_H_6_)HgCl_3_ contains the π-conjugated planar ligand melamine, which adopts a nearly coplanar orientation with a small dihedral angle of 2.983°, preventing antiparallel stacking and establishing the structural basis for a 5.0 × KDP SHG response. But these layers, connected only by weak N–H⋯N and N–H⋯Cl hydrogen bonds, result in a lower thermal decomposition temperature (225 °C);^20^ H_11_C_4_N_2_CdI_3_ contains oriented [CdNI_3_] tetrahedra that underpin a 6.0 × KDP SHG response. Nevertheless, the framework is mainly sustained by weak Cd–I coordination and hydrogen bonds, leading to a decomposition temperature of 300 °C.^21^ On the other hand, enhancing metal–ligand coordination and constructing dense frameworks can improve stability, but often induce centrosymmetric or racemic packing, causing dipole cancellation and suppressing NLO responses.^22–27^ [Cd(BPHY)(SA)]_*n*_ achieves a decomposition temperature of 350 °C, driven by strong Cd–O/N coordination and a densely packed framework. Unfortunately, the centrosymmetric 2D [Cd(SA)]_*n*_ layers induce partial dipole-moment cancellation, and the resulting 3D assembly with BPHY fails to achieve cooperative alignment of polar units, yielding a weak SHG response of 0.1 × KDP;^28^ [Zn(Mitz)Cl]_*n*_ is dominated by Zn–N coordination, with Mitz ligands acting as three-connected nodes bridging Zn^2+^ centers to form a rigid and robust framework, resulting in a decomposition temperature exceeding 400 °C. Yet, its racemic structure composed of left and right-handed helical chains causes partial dipole cancellation, limiting its SHG response to only 2.0 × KDP;^29^ Cd(AmTAZ)Cl forms a dense 3D network through strong Cd–N coordination, reaching a decomposition temperature of 380 °C. Conversely, the crystal exhibits inversion twinning (Flack parameter: 0.51), and this racemic nature leads to dipole cancellation, making the SHG response lower than that of KDP.^30^ Therefore, how to simultaneously achieve strong NLO activity and high stability in MOCs has not yet been systematically investigated, representing a key issue this study aims to address.

Here, we propose a “polar-layer-dense-locking” strategy (Fig. 1b), which consists of two key steps: (1) construction of nonlinear layers: a rigid organic ligand with high molecular hyperpolarizability is chosen as the “nonlinear source”, and its coordinating atoms bridge metal ions to assemble polar two-dimensional layers, enabling the cooperative alignment of ligand dipoles and efficiently translating microscopic hyperpolarizability into a macroscopic SHG response; (2) stability locking: the multidentate bridging capability of the ligand links polarized polyhedra into a three-dimensional topological network *via* strong coordination bonds, thereby conferring high structural stability while retaining strong SHG. This strategy is expected to provide a scalable approach that simultaneously achieves strong second harmonic generation and high structural stability. Based on this strategy, imidazole-4-carboxylate (imc^2−^) was selected as the ligand. As shown in Fig. 1c, density functional theory (DFT) calculations indicate that imc^2−^ possesses significantly higher hyperpolarizability than representative N-containing rigid planar π-conjugated units, such as pyridine, pyrazole, thiazole, and pyridazine (aug-cc-pVTZ) (Table S1).^31^ The angle between the carboxyl group and the imidazole nitrogen atom is close to 120°, favoring a µ_2_-κ^2^-N, O bridging mode. This is expected to construct polar 2D layers with well-defined directionality, which are further assembled into a robust 3D polar topological framework through strong metal–ligand coordination. In this work, a novel 3D metal–organic complex (MOC), Cd(imc) (imc^2−^ = imidazole-4-carboxylate), was successfully synthesized.

This material exhibits excellent comprehensive performance, including a strong SHG response (10.0 × KDP), high thermal stability (TG = 470 °C), and exceptional water resistance (stable in water at room temperature for > 180 days and at 100 °C for >30 days), as well as a wide band gap (4.28 eV). Theoretical calculations also confirm that the imc^2−^ ligand contributes 91% of the total SHG effect. This work employs µ_2_-κ^2^-N, O bridging to construct high-performance 2D layers, promoting the cooperative alignment of ligand dipoles and effectively converting microscopic high hyperpolarizability into a strong SHG response. Strong Cd–O coordination and dense interlayer packing are then utilized to assemble a 3D topological network, ensuring the simultaneous achievement of robust SHG and high structural stability. These results elucidate the cooperative mechanism of the “polar-layer-dense-locking” strategy, demonstrating that this crystal engineering approach can effectively address the SHG-stability trade-off in MOCs-based NLO materials and provide a reproducible paradigm for the design of high-performance UV crystals.

## Results and discussion

Yellow transparent Cd(imc) bulk crystals were synthesized using the conventional solvothermal method. With a yield of approximately 52% (based on Cd) (Fig. S1). For detailed synthetic methods, please refer to the SI. Comprehensive crystallographic data, atomic coordinates, and bonding information for this structure are listed in Table S2–S8. The phase purity of the compound was verified by powder X-ray diffraction (PXRD) (Fig. S2). Field-emission scanning electron microscopy (FESEM) combined with energy-dispersive X-ray spectroscopy (EDS) confirmed the presence and uniform distribution of C, N, O, and Cd (Fig. S3).

**Fig. 2:**
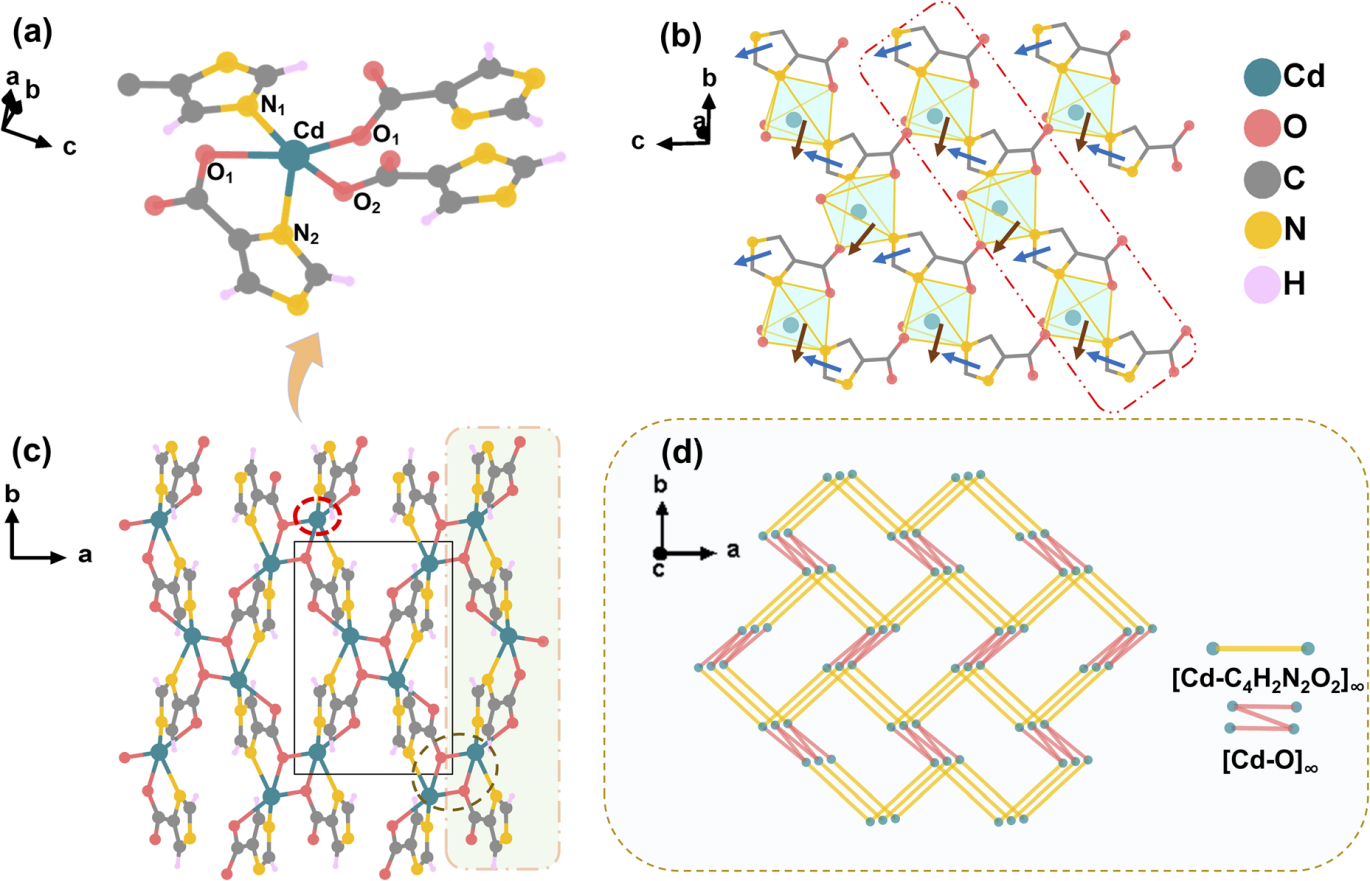
(a) Coordination environment of the central metal Cd. (b) 1D chain (red box) and 2D layer structure along the *bc* plane. (Blue arrows correspond to the dipole moments of imc^2−^ ligands, and brown arrows denote those of the Cd coordination polyhedra) (c) 3D framework structure viewed along the crystallographic *ab* plane, demonstrating the interlayer linkage of 2D layers *via* infinite [Cd–O]_∞_ coordination chains. (d) Simplified structure of the 3D topological network.

The crystal structure was determined by single-crystal X-ray diffraction. It crystallizes in the orthorhombic crystal system with a polar space group of *Pna*2_1_ (No. 33). The unit–cell parameters are as follows: *a* = 6.9584(6) Å, *b* = 10.2697(9) Å, *c* = 6.8280(6) Å, *α* = *β* = *γ* = 90°, *Z* = 4, *V* = 487.93(7) Å^3^. As shown in Fig. 2a, the central Cd^2+^ ion is coordinated by four imc^2−^ ligands. Specifically, one ligand contributes one N atom, one ligand chelates through one N and one O atom, and the remaining two ligands each donate one O atom, collectively forming an irregular five-coordinate [CdO_3_N_2_] polyhedron.

The Cd–O bond lengths are 2.234(5) Å, 2.361(4) Å, and 2.529(5) Å, while the Cd–N bond lengths are 2.161(6) Å and 2.216(5) Å (Fig. S4). The bond angles within this coordination polyhedron range from 70.51(17)° to 147.09(15)°. Along the *bc* plane, the [CdO_3_N_2_] polyhedra are linked *via* bidentate coordination to two N atoms of the imc^2−^ ligands, forming one-dimensional chains (red box in Fig. 2b). The two O atoms of the carboxyl group in the ligand coordinate with Cd^2+^ to form [Cd–COO^−^–Cd] chains, which link the one-dimensional chains into rigid two-dimensional layers (Fig. 2b). This effectively constrains the ligand orientation, promoting the parallel alignment of molecular hyperpolarizability within the lattice. Along the *b*-axis, the dihedral angle between two adjacent imc^2−^ ligands is close to 40° (Fig. S5), which effectively prevents the mutual cancellation of dipole moments within the layer. In the 2D layers, the rigid framework constructed *via* bidentate coordination promotes the directional alignment of organic ligand dipoles (Fig. S6 and S7), which significantly facilitates the effective superposition of the second-order nonlinear polarizability. Adjacent 2D layers are further interwoven through infinite [Cd–O]_∞_ chains, ultimately forming a 3D network structure (Fig. 2c). To more intuitively display the 3D network of this complex, the structure is simplified into the 3D topological network shown in Fig. 2d.

**Fig. 3:**
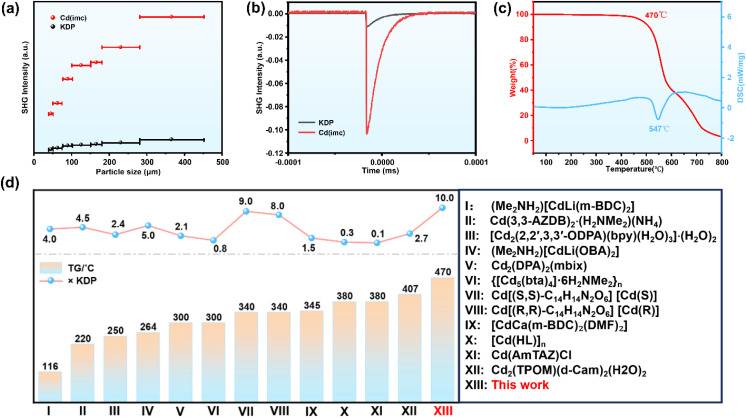
(a) SHG intensity *versus* particle size under 1064 nm laser irradiation. (b) Relationship between SHG intensity and particle size under 1064 nm laser irradiation. (c) TGA-DSC curves. (d) Comparison of SHG intensity and thermal stability between Cd(imc) and other cadmium-based 3D metal–organic complexes (including some H_2_O-containing complexes, where structural and thermal analyses in the original text confirmed that water molecules have no significant effect on the stability of the framework structure).

**Fig. 4:**
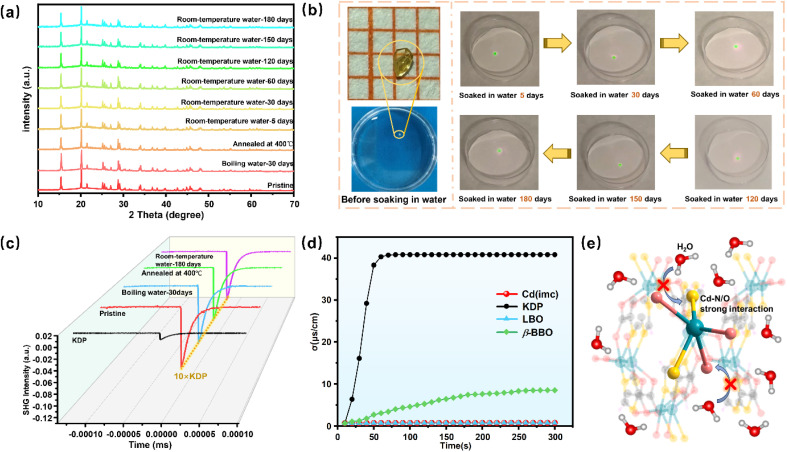
(a) PXRD patterns after treatment under different conditions. (b) Water resistance test of the as-synthesized Cd(imc) crystals (the crystals emit green light at 532 nm when irradiated with a 1064 nm laser). (c) SHG intensity response after treatment under different conditions. (d) Conductivity changes of Cd(imc), KDP, β-BBO, and LBO crystals in water. (e) Schematic illustration of the water-resistance structural mechanism of Cd(imc).

To further validate the structural rationality of Cd(imc), infrared (IR) spectroscopy was performed on this compound (Fig. S8). The absorption band at 3133 cm^−1^ is assigned to the stretching vibration of the C–H bonds on the imidazole ring. The bands at 1540 and 1386 cm^−1^ correspond to the asymmetric and symmetric stretching vibrations of COO^−^, respectively, indicating complete deprotonation of the carboxyl group. The absorption at 1697 cm^−1^ can be attributed to the C=O stretching vibration in the carboxylate moiety. The absorption bands in the 1220–1112 cm^−1^ range are assigned to the stretching vibrations of C–N and C–O. In addition, the bands at 779–821 cm^−1^ correspond to the out-of-plane bending vibrations of the imidazole C–H bonds, while those at 565–661 cm^−1^ are assigned to the stretching vibrations of Cd–O/N, urther confirming the coordination between the imc^2−^ ligands and Cd^2+^ ions.^32,33^ Additionally, to explore the optical properties of this compound, UV-vis diffuse reflectance spectroscopy was conducted (Fig. S9). Based on the Kubelka–Munk equation, the experimental optical band gap was determined to be 4.28 eV. Notably, Cd(imc) exhibits excellent nonlinear optical performance as well as high stability. The SHG response of Cd(imc) at 1064 nm was measured using the Kurtz-Perry powder technique.^34^ The SHG intensity of Cd(imc) gradually increases with particle size and reaches a plateau in the 280–450 µm range, indicating that the material satisfies the phase-matching condition (Fig. 3a). Specifically, at the same platform particle size (280–450 µm), the SHG intensity of Cd(imc) is approximately 10 times that of KDP powder (Fig. 3b). Simultaneously, TGA-DSC analysis shows that Cd(imc) begins to decompose at approximately 470 °C, with no phase transition observed prior to decomposition (Fig. 3c), indicating its high thermal stability. To objectively evaluate the comprehensive performance of Cd(imc), we conducted a comparative analysis of its SHG intensity and thermal decomposition temperature with those of previously reported Cd-based 3D-MOCs (Fig. 3d and Table S9). The result reveals that Cd(imc) simultaneously exhibiting high SHG (>10.0 × KDP) and high thermal stability (TG > 400 °C), which are extremely rare in the 3D-MOCs. To further investigate the stability of Cd(imc), the samples were subjected to various treatments: immersion in boiling water (100 °C) for 30 days, annealing at 400 °C for 1 h, and soaking in water at room temperature for 180 days. The PXRD patterns of the treated samples remain largely consistent with that of the as-synthesized compound (Fig. 4a), indicating that the structure of Cd(imc) is preserved under the above conditions. Subsequently, we irradiated the samples treated with room-temperature water using a 1064 nm laser, all of which exhibited strong green emission (Fig. 4b). Powder SHG tests further show that the SHG intensities remain at approximately 10.0 × KDP (Fig. 4c), demonstrating that the nonlinear optical response remains unaffected even after prolonged exposure to water and high temperatures. To further verify the excellent water resistance of Cd(imc), equal masses of Cd(imc), KDP, β-BBO, and LBO were each immersed in equal volumes of ultrapure water, and the change in solution conductivity over time was monitored (Fig. 4d). The results show that the conductivity of the KDP solution increases sharply within a few seconds and reaches equilibrium in approximately 1 minute, consistent with its high water solubility.^35^ For β-BBO, the conductivity exhibits a gradual upward trend due to its slight solubility in water.^36^ In contrast, the conductivities of the Cd(imc) and LBO solutions remained nearly constant over the entire testing period, indicating negligible ion leaching into the aqueous phase. Considering that LBO is a well-known inorganic NLO crystal with high chemical stability and excellent water resistance,^37^ this result further demonstrates that Cd(imc) possesses water resistance comparable to that of highly stable inorganic materials. As shown in Fig. 4e, the dense and highly cross-linked 3D polar framework of Cd(imc) is formed through µ_2_-bridging coordination between the N/O sites of the ligand and Cd^2+^ ions, further reinforced by strong interlayer Cd–O coordination. Within this compact coordination network, the strong covalent Cd–N/O interactions confer high thermodynamic stability upon the framework. On the other hand, the topological network creates significant steric hindrance, which effectively impedes the nucleophilic attack of water molecules on the metal centers, thereby leading to strong water resistance.

To elucidate the structure–property relationship of Cd(imc), we approached the study from the perspective of ligand design, systematically dissecting the correlation between its assembly mechanism and functional properties. During the hydrothermal synthesis, the addition of KOH induces the simultaneous deprotonation of the carboxyl and imidazole groups, thereby activating all coordination sites of the imc^2−^ ligand (namely the two nitrogen atoms of the imidazole ring and the two oxygen atoms of the carboxylate group). Upon deprotonation, the pyrrolic N–H of the imidazole ring loses a proton to form an N^−^ species, endowing both nitrogen atoms with stable σ-don or ability. The ligand coordinates to Cd^2+^ ions *via* a µ_2_-κ^2^-N^1^, N^2^ mode, forming one-dimensional chains. This bidentate coordination prevents potential chain breaks associated with monodentate binding, thereby maintaining the structural integrity of the chain segments. Simultaneously, the fully deprotonated carboxylate groups (–COO^−^) connect adjacent 1D chains *via* a µ_2_-κ^2^-N^1^, N^2^ bidentate coordination mode, fixing them into rigid two-dimensional layers. The synergistic N, O multidentate bridging enforces a uniform orientation of the [CdO_3_N_2_] polyhedra and organic ligands within the 2D layers. The rigid five-membered chelate ring (Cd–N–C–C–O) formed *via* coordination restricts rotation of single bonds within the ring, effectively anchoring both ligands and metal centers. This arrangement facilitates the effective superposition of dipole moments along a specific crystallographic axis, forming the structural basis for the strong SHG response. The 2D layers are further linked into a compact 3D framework through strong interlayer Cd–O coordination bonds. The coordination-saturating effect of Cd^2+^ ions reduces structural voids, thereby enhancing the framework stability and rendering it resistant to disruption.

**Fig. 5:**
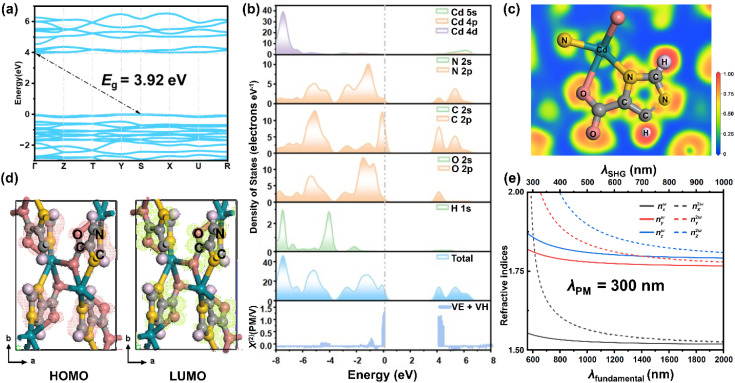
(a) Band structure. (b) Total DOS and orbital-resolved partial DOS. (c) Projected electron localization function (ELF). (d) Frontier orbital distributio[001]. (e) Refractive index dispersion curves with the shortest phase-matching wavelength *λ*_PM_ = 300 nm.

To understand the relationship between structure and optical properties, first-principles calculations were performed. As shown in Fig. 5a, the calculated band gap is 3.94 eV, slightly smaller than the experimental value of 4.28 eV. This discrepancy primarily arises from the inherent underestimation of the exchange–correlation energy by the GGA functional.^38^ The total and partial density of states (DOS/PDOS) (Fig. 5b) reveal that within the range of −8 to 8 eV, the valence band maximum (VBM) is predominantly composed of the N 2p, C 2p, and O 2p orbitals of the imc^2−^ ligand, with minor contributions from the Cd 4d orbitals. The conduction band minimum (CBM) is also dominated by the N 2p, C 2p, and O 2p orbitals of the ligand, indicating that π → π* excitations within the organic backbone are the primary source of the optical transitions. Furthermore, the calculated electron localization function (ELF) (Fig. 5c) shows that the π-electrons delocalized along the conjugated imidazole orbitals. Combined with the analysis of the frontier molecular backbone exhibit a high degree of localization, indicating strong π-covalency and significant delocalization of the πorbitals (Fig. 5d), the highest occupied molecular orbital-lowest unoccupied molecular orbital (HOMO–LUMO) electron transitions occur primarily on the C, N, and O atoms of the imc^2−^ ligand.

Cd(imc) crystallizes in a negative biaxial system, satisfying *n*_c_ > *n*_b_ > *n*_a_, with a calculated birefringence of 0.32 @ 546 nm (Fig. S10). Polarized optical microscopy measurements at 546 nm give a birefringence of 0.31 (Fig. S11), in good agreement with the calculated value. The refractive index dispersion curves (Fig. 5e) indicate that the shortest phase-matching wavelength for SHG (*λ*_PM_) is approximately 300 nm.

**Fig. 6:**
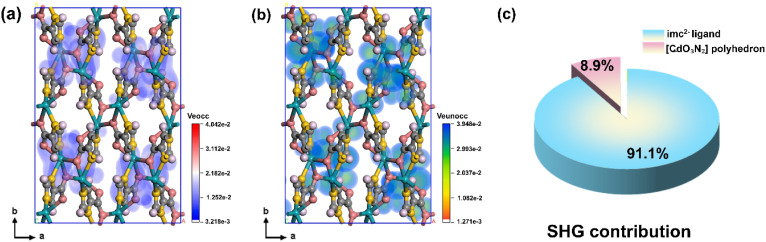
(a and b) SHG-weighted electron density from real-space atom cutting (Veocc and Veunocc). (c) Percentage contribution to the SHG response.

Based on Kleinman symmetry, the nonzero second-order NLO tensor components of Cd(imc) (point group *mm*2) are *d*_15_ = 4.20 pm V^−1^, *d*_24_ = −3.99 pm V^−1^ and *d*_33_ = 11.19 pm V^−1^. Notably, for phase-matched SHG in the *mm*2 point group, the dominant contributions arise from the effective tensor components rather than the non-phase-matched *d*_33_.^39^ In summary, the calculated effective nonlinear coefficient (*d*_eff_) is 3.78 pm V^−1^, corresponding to a theoretical SHG response of 9.69 × KDP (*d*_36_ = 0.39 pm V^−1^), which is in good agreement with the experimental value of 10.0 × KDP. The microscopic origin of the SHG response was quantitatively analyzed by calculating the dipole moments, polarizabilities, and hyperpolarizabilities of the Cd-centered coordination polyhedra and imc^2−^ ligands in Cd(imc) (Table S10), and further examined using the real-space atom-cutting technique combined with SHG-weighted electron density analysis (Fig. 6a, b and Table S11).^40,41^ The imc^2−^ ligands contribute 91.1% of the total SHG response, whereas the [CdO_3_N_2_] polyhedra account for only 8.9%, confirming that the ligand hyperpolarizability dominates the microscopic nonlinearity, while the oriented alignment of [CdO_3_N_2_] polyhedra and organic ligands amplifies the macroscopic *χ*^(2)^ (Fig. 6c).

## Conclusions

Based on a crystal engineering-oriented “polar-layer-dense-locking” strategy, this study successfully designed and synthesized a novel 3D cadmium-based complex, Cd(imc), achieving a rare combination of strong SHG response and high stability in a MOCs. It exhibits a strong SHG response (10.0 × KDP), a high decomposition temperature (470 °C), a good water resistance (stable in water at room temperature for over 180 days and in boiling water for more than 30 days), as well as a wide optical band gap of 4.28 eV, making it a promising candidate for UV NLO applications. Structural analysis and theoretical calculations indicate that the π-conjugated system of the imc^2−^ ligand contributes approximately 91% of the SHG response, while the µ_2_-N, O bidentate bridging forms rigid 2D layers that enforce directional alignment of dipoles, ensuring a strong SHG response; the strong Cd–O coordination bonds and the tightly cross-linked 3D network effectively hinder water molecule infiltration, ensuring the high stability. This work not only provides a high-performance UV NLO candidate, but also demonstrates the feasibility of the “polar-layer-dense-locking” strategy for simultaneously achieving strong SHG response and high stability in MOCs. It offers a reference framework for the design of UV-MOCs-NLO materials and presents a new avenue for advancing the development of MOCs in the NLO field.

## Author contributions

Jiaqi Qian: formal analysis, writing – original draft, writing – review & editing. Yunjie Wang: data curation, formal analysis. Jiafu Ding: theoretical calculations. Xin Su: methodology authorization, resource provision. Qi Wu: funding acquisition, supervision, conceptualization, writing – review & editing.

## Conflicts of interest

There are no conflicts to declare.

## Supplementary Material

SC-OLF-D6SC03803D-s001

SC-OLF-D6SC03803D-s002

## Data Availability

CCDC 2522036 contains the supplementary crystallographic data for this paper.^42^ The data that support the findings of this study are available in the supplementary information (SI) of this article. Supplementary information: experimental section and additional tables and figures. See DOI: https://doi.org/10.1039/d6sc03803d.
