# Supplementary material for: Breaking the SHG-stability trade-off in UV nonlinear optical materials by polar-layer-dense-locking
Source: Chem Sci. 2026 May 29;17(27):13691–8. doi: 10.1039/d6sc03803d (PMC13238906; doi:10.1039/d6sc03803d)
Supplement: SC-017-D6SC03803D-s001 [file SC-017-D6SC03803D-s001.pdf]

Supplementary Information (SI) for Chemical Science.
This journal is © The Royal Society of Chemistry 2026

Supporting Information

Breaking the SHG-Stability Trade-off in UV Nonlinear Optical Materials by Polar-Layer-Dense-Locking

Jiaqi Qian, [a] Yunjie Wang, [a] Jiafu Ding, [b] Xin Su, [b] and Qi Wu *[a]

[a] State Key Laboratory of New Textile Materials and Advanced Processing Technologies, Wuhan Textile University Wuhan 430200, China, E-mail: wuqi2011@whu.edu.cn
[b] Xinjiang Laboratory of Phase Transitions and Microstructures in Condensed Matter Physics, College of Physical Science and Technology, Yili-Normal University, Xinjiang 835000, China.

Contents

Experimental Procedures 3
Results and Discussion 4
Table S1. Comparison of the hyperpolarizability (lβ_{max}l), HOMO-LUMO energy gap (E_g), dipole moment (μ), and polarizability (α) of imc$^-$ and representative N-containing rigid planar π-conjugated ligands. 5
Table S2. Crystal data and structure refinement for Cd(imc) 6
Table S3. Atomic coordinates and equivalent isotropic displacement parameters and occupancies of Cd(imc). 7
Table S4. Anisotropic displacement parameters (Å^2×10^3) for Cd(imc). 7
Table S5. Bond lengths for Cd(imc). 7
Table S6. Bond angles for Cd(imc). 8
Table S7. Atomic coordinates and equivalent isotropic displacement parameters and occupancies of Cd(imc) 8
Table S8. Anisotropic displacement parameters (Å^2×10^3) for Cd(imc). 8
Table S9. Statistics of SHG Intensity and TG of Cd-Based 3D Metal-Organic Complexes 9
Table S10. Calculated hyperpolarizability, distortion index and dipole moment of compounds Cd(imc). 9
Table S11. The SHG contribution of the compound Cd(imc) after RSAC treatment 9
Fig. S1. Color comparison of crystals and powder. 10
Fig. S2. Experimental and calculated powder XRD patterns of Cd(imc) 10
Fig. S3. Scanning electron microscope image of Cd(imc) and its elemental distribution… 11
Fig. S4. Coordination environment of Cd^{2+} in Cd(imc) 11
Fig. S5. The dihedral angle between the organic rings in Cd(imc) 11
Fig. S6. Dipole moment orientations in different planes of Cd(imc) 12
Fig. S7. Directions of the non-zero SHG tensor components in Cd(imc) 12
Fig. S8. IR spectra of Cd(imc) 13
Fig. S9. UV-Vis diffuse reflectance spectra of Cd(imc) 13
Fig. S10. Theoretically calculated birefringence of Cd(imc) 14
Fig. S11. Photographs of the tested Cd(imc) crystals for birefringence 14
References 15

Experimental Procedures

Reagents

1H-imidazole-4-carboxylic acid ($C_4H_4N_2O_2$, 98%, Adamas), cadmium chloride ($CdCl_2$, 99%, macklin), potassium hydroxide (KOH, 95%, macklin). They are all used without further purification.

Synthesis

The coordination compound Cd(imc) was synthesized by a solvothermal method. 1H-imidazole-4-carboxylic acid (0.224 g, 2 mmol), $CdCl_2$ (0.366 g, 2 mmol),KOH(0.112 g, 2 mmol) and deionized water (6 mL) were successively added into a 25 mL Teflon-lined autoclave, which was then sealed and heated in an oven at 180 °C for 24 h. After the reaction, the autoclave was slowly cooled to room temperature at a rate of 0.10 °C·min^{-1}, affording yellow block-shaped crystals of Cd(imc) with a yield of 52%.

Single-crystal X-ray diffraction

The single-crystal X-ray diffraction (SC-XRD) data for Cd(imc) were collected on an XtaLAB Synergy R equipped with a graphite monochromator using Mo Kα radiation (λ = 0.71073 Å) at 293 K. The crystal structure was solved by Direct Methods with Olex2 and refined by least-squares techniques on F^2 with anisotropic thermal parameters for all atoms.[1, 2] Crystal data and structure refinement information for Cd(imc) are summarized in Table S2. Atomic coordinates and equivalent isotropic displacement parameters, anisotropic displacement parameters, selected bond distances and angles are collected in Table S3-S6.

Powder X-ray Diffraction (PXRD)

Powder X-ray diffraction data were recorded on an Advance diffractometer at A temperature of 40 kV, 100 mA, Cu Kα1 radiation (Bruker D2, radiation wave number 1.5406 A), scanning speed of 0.5°/min, and scanning angular range of 10-70°. The phase purity was determined by powder X-ray diffraction.

EDS

The elemental distribution maps and microprobe elemental analyses were measured on a field-emission scanning electron microscope (SEM, JSM-7800) equipped with an energy dispersive X-ray spectroscope (EDS, Oxford INCA).

Infrared Spectrum

The IR spectra was collected on a Nicolet iS5 Fourier-transformed infrared spectrometer at room temperature in the wavelength range of 4000-400 cm^{-1}. The sample and dry KBr are mixed and ground in a certain proportion and pressed into flakes for measurement. (weight ratio = 1:100).

UV-vis Diffuse Reflectance Spectroscopy

The UV-vis diffuse reflectance spectra were obtained by using a Varian Cary 5000 spectrophotometer with a scan range of 200-800 nm at room temperature. The spectrally pure barium sulfate was selected as a reference (100% reflectance), and a ground powder sample was coated on its surface for testing.

Thermal analysis

Thermogravimetric-differential scanning calorimetry (TGA-DSC) measurements were carried out on a Netzsch STA 449 F5 thermal analyzer. Approximately 0.8 mg of the sample was placed in a platinum crucible and heated from room temperature to 800 °C at a heating rate of 10 K min^{-1} under a nitrogen atmosphere.

Water resistance test

The conductivity of sample solutions was recorded in a Az8303 portable conductivity meter. 1 mg of Cd(imc), KDP, *β*-BBO and LBO were injected into to a beaker containing 50 mL of ultrapure water respectively, and then record the conductivity of the solution per 10 seconds using the conductivity meter.

Second Harmonic Generation (SHG) Measurement

The second-order NLO measurements of the powder samples were performed at room temperature, and the SHG effect of the powder samples was measured using a Q-switched 1064 nm Nd: YAG laser by using the improved Kurtz and Perry methods. Polycrystalline samples of this compound were sieved into different particle sizes (35-50, 50-74, 74-100, 100-154, 154-180, 180-280 and 280-450 μm) to investigate whether its SHG response could be phase matched. The reference substance KH_2PO_4 (KDP) was selected to compare with the SHG efficiency of the sample, so as to evaluate the second-order NLO effect of the measured sample.

Computational method

The electronic structure and optical properties of compounds Cd(imc) were calculated using CASTEP software, and the geometric structure optimization of the system is carried out by density functional theory.[3] The Perdew-Burke-Ernzerhof (PBE) functional (under the Generalized Gradient Approximation, GGA) was chosen for the exchange-correlation term., with norm-conserving pseudopotentials (NCP) employed to describe the interaction potential between ionic cores and valence electrons.[4] To ensure computational convergence, the plane wave cutoff energy under NCP is set to 910 eV for Cd(imc). K-points are sampled using the Monkhorst-Pack method with an interval of 0.04 $Å^{-1}$.[5] Self-consistent calculations require the total energy convergence tolerance of the system to be less than 1×10^{-6} eV/atom, the force on a single atom to be less than 0.01 eV/Å, the maximum atomic displacement tolerance to be less than 5×10^{-4} nm, and the maximum stress deviation to be less than 0.02 GPa. The ion-electron interactions of all atoms are modeled by the NCP, and the atomic electron configurations are: H($1s^1$), C($2s^2 2p^2$), N($2s^2 2p^3$), O($2s^2 2p^4$), Cl($3s^2 3p^5$), Cd($4d^{10} 4p^6 5s^2$).

The highest occupied molecular orbital (HOMO) and the lowest unoccupied molecular orbital (LUMO) of Cd(imc) were calculated using Gaussian 09W software, and the static polarization rate anisotropy (δ) and maximum hyperpolarization rate tensor ($|\beta_{max}|$) were calculated using the LanL2DZ base group of mixed functional B3LYP and density functional theory.[6, 7]

To quantify the degree of aberration of these single hexahedral units, the eccentric aberration change index (Ɗ) is calculated using the following equations:

$$Ɗ = \sum_{i=1}^{3} \frac{|a_i - b_i|}{a_i + b_i}$$

where a_i and b_i are the short and long Cd-X/Y bond lengths.

To gain insight into the contributions of each component in the crystal to the SHG effect and birefringence, we employed the real-space atomic cutting method. The specific procedures are as follows: the atomic cutting radius was set to half the average bond length of each atom, yielding cutting radii of H-0.37 Å, C-0.77 Å, N-0.74 Å, O-1.10 Å, and Cd-1.41 Å. Finally, the constituent groups of the crystal were divided into two parts, namely cut-Cd and cut-CN.[8, 9]

The polarizability anisotropy was calculated using Gaussian 09 at the aug-cc-PVTZ level.

Results and Discussion

Table S1. Comparison of the hyperpolarizability (lβ_{max}l), HOMO-LUMO energy gap (E_g), dipole moment (μ), and polarizability (α) of imc$^-$ and representative N-containing rigid planar π-conjugated ligands.

Compounds	Chemical Formula	Hyperpolarizability (|β_{max}|)	HOMO-LUMO gap(E_g)	Dipole Moment (μ)	Polarizability (α)
Pyrazine	$C_4H_4N_2$	0.230	4.448	0.009	56.553
1,3,5-triazine	$C_3H_3N_3$	0.834	4.447	0.040	48.449
Oxazole	C_3H_3NO	28.228	4.866	1.505	37.792
Pyrrole	C_4H_5N	28.998	6.738	2.015	31.934
Thiazole	C_3H_3NS	32.564	6.227	2.740	41.043
Pyrimidine	$C_4H_4N_2$	45.825	4.397	2.721	53.859
Pyridine	C_5H_5N	47.197	5.145	2.736	58.495
1H-prazole	$C_3H_4N_2$	57.662	5.228	2.319	43.116
Pyridazine	$C_4H_4N_2$	88.204	4.699	5.005	54.800
1H-imidazole	$C_3H_4N_2$	116.660	5.243	3.978	42.341
**imc^{2-}**	**$C_4H_2N_2O_2$**	**202.720**	**5.253**	**11.300**	**40.830**

Table S2. Crystal data and structure refinement for Cd(imc).

Compound	Cd(imc)
Empirical formula	$C_4H_2CdN_2O_2$
Formula weight	222.48
Temperature/K	304.00
Crystal system	orthorhombic
Space group	$Pna2_1$
a/Å	6.9584(6)
b/Å	10.2697(9)
c/Å	6.8280(6)
α/°	90
β/°	90
γ/°	90
Volume/Å^3	487.93(7)
Z	4
ρ_{calc}g/cm^3	3.029
μ/mm^{-1}	4.371
F(000)	416.0
2Θ range for data collection/°	7.074 to 51.916
Index ranges	$-8 \le h \le 8$, $-12 \le k \le 12$, $-8 \le l \le 8$
Reflections collected	7537
Independent reflections	952 [R_{int} = 0.0382, R_{sigma} = 0.0261]
Goodness-of-fit on F^2	1.214
Final R indexes [I>=2σ (I)]	R_1 = 0.0221, wR_2 = 0.0439
Final R indexes [all data]	R_1 = 0.0251, wR_2 = 0.0451
Largest diff. peak/hole / e Å^3	0.41/-0.50
Flack parameter	0.05(3)
CCDC number	2522036

[a] $R_1 = \sum||F_o| - |F_c||/\sum|F_o|$; $wR_2 = [\sum w(F_o^2 - F_c^2)^2]/\sum w(F_o^2)^2]^{1/2}$

Table S3. Atomic coordinates and equivalent isotropic displacement parameters and occupancies of Cd(imc).

Atom	*x*	*y*	*z*	U_{eq} (Å^2)
Cd1	8554.5(5)	958.7(3)	817.9(12)	16.10(16)
N1	8393(7)	2263(5)	3296(8)	16.0(12)
O2	5728(7)	4288(4)	8439(7)	20.9(11)
O1	6198(6)	2209(4)	9187(7)	21.5(11)
C3	7357(8)	3012(6)	6144(10)	11.7(17)
N2	7766(8)	4072(5)	5001(8)	17.1(12)
C2	7760(9)	1918(6)	5113(9)	14.6(14)
C1	8377(9)	3564(7)	3306(11)	20.0(15)
C4	6408(8)	3153(7)	8061(10)	16.4(15)

Table S4. Anisotropic displacement parameters (Å^2×10^3) for Cd(imc). The anisotropic displacement factor exponent takes the form: -2π^2[$h^2a^{*2}U_{11}$+2hka*b*U_{12}+…].

Atom	U_{11}	U_{22}	U_{33}	U_{23}	U_{13}	U_{12}
Cd1	23.3(2)	13.1(2)	11.9(2)	-2.3(4)	2.6(5)	-0.39(17)
N1	18(3)	15(3)	14(3)	-3(3)	0(2)	1(2)
O2	26(3)	17(2)	20(3)	-2(2)	8(2)	3(2)
O1	27(3)	23(3)	15(2)	9(2)	-1.2(19)	0(2)
C3	13(3)	12(3)	10(5)	2(3)	-1(3)	3(2)
N2	18(3)	17(3)	16(3)	0(2)	3(2)	3(2)
C2	14(3)	14(3)	16(3)	3(3)	2(2)	8(3)
C1	25(4)	21(4)	14(4)	2(3)	2(3)	3(3)
C4	16(4)	18(4)	14(3)	-3(3)	-4(3)	0(3)

Table S5. Bond lengths for Cd(imc).

Atom	Atom	Length/Å	Atom	Atom	Length/Å
Cd1	N1	2.161(6)	O2	O2	1.284(8)
Cd1	O2^1	2.234(5)	O1	O1	1.246(8)
Cd1	O2^2	2.529(5)	C3	C3	1.369(8)
Cd1	O1^3	2.361(4)	C3	C3	1.356(8)
Cd1	N2^2	2.216(5)	C3	C3	1.473(10)
N1	C2	1.363(8)	N2	N2	1.339(9)
N1	C1	1.336(9)			

^{1}1/2+*X*,1/2-*Y*, -1+*Z*; ^{2}3/2-*X*, -1/2+*Y*, -1/2+*Z*; ^{3}+*X*, +*Y*, -1+*Z*

Table S6. Bond angles for Cd(imc).

Atom	Atom	Atom	Angle/°	Atom	Atom	Atom	Angle/°
N1	Cd1	O2[1]	132.43(18)	C4	O2	Cd1[5]	113.6(4)
N1	Cd1	O2[2]	82.93(18)	C4	O2	Cd1[4]	107.0(4)
N1	Cd1	O1[3]	89.78(18)	C4	O1	Cd1[6]	129.3(4)
N1	Cd1	N2[2]	135.8(2)	N2	C3	C4	121.5(6)
O2[1]	Cd1	O2[2]	107.70(6)	C2	C3	N2	108.7(6)
O2[1]	Cd1	O1[3]	100.88(17)	C2	C3	C4	129.4(6)
O1[3]	Cd1	O2[2]	147.09(15)	C3	N2	Cd1[5]	117.7(4)
N2[2]	Cd1	O2[2]	70.51(17)	C1	N2	Cd1[5]	133.6(5)
N2[2]	Cd1	O2[1]	89.93(19)	C1	N2	C3	104.4(6)
N2[2]	Cd1	O1[3]	93.94(17)	C3	C2	N1	108.9(6)
C2	N1	Cd1	124.6(4)	N1	C1	N2	113.4(7)
C1	N1	Cd1	128.6(5)	O2	C4	C3	115.6(6)
C1	N1	C2	104.6(6)	O1	C4	O2	122.6(6)
Cd1[4]	O2	Cd1[5]	136.5(2)	O1	C4	C3	121.6(6)

[1]1/2+*X*,1/2-*Y*, -1+*Z*; [2]3/2-*X*, -1/2+*Y*, -1/2+*Z*; [3]+*X*, +*Y*, -1+*Z*;[4]-1/2+*X*,1/2-*Y*,1+*Z*; [5]3/2-*X*,1/2+*Y*,1/2+*Z*; 6+*X*, +*Y*,1+*Z*

Table S7. Atomic coordinates and equivalent isotropic displacement parameters and occupancies of Cd(imc).

Atom	*x*	*y*	*z*	U_{eq} (Å^2)
Cd1	8554.5(5)	958.7(3)	817.9(12)	16.10(16)
N1	8393(7)	2263(5)	3296(8)	16.0(12)
O2	5728(7)	4288(4)	8439(7)	20.9(11)
O1	6198(6)	2209(4)	9187(7)	21.5(11)
C3	7357(8)	3012(6)	6144(10)	11.7(17)
N2	7766(8)	4072(5)	5001(8)	17.1(12)
C2	7760(9)	1918(6)	5113(9)	14.6(14)

Table S8. Anisotropic displacement parameters (Å^2×10^3) for Cd(imc). The anisotropic displacement factor exponent takes the form: $-2\pi^2[h^2a^{*2}U_{11}+2hka^*b^*U_{12}+\ldots]$.

Atom	U_{11}	U_{22}	U_{33}	U_{23}	U_{13}	U_{12}
Cd1	23.3(2)	13.1(2)	11.9(2)	-2.3(4)	2.6(5)	-0.39(17)
N1	18(3)	15(3)	14(3)	-3(3)	0(2)	1(2)
O2	26(3)	17(2)	20(3)	-2(2)	8(2)	3(2)
O1	27(3)	23(3)	15(2)	9(2)	-1.2(19)	0(2)
C3	13(3)	12(3)	10(5)	2(3)	-1(3)	3(2)
N2	18(3)	17(3)	16(3)	0(2)	3(2)	3(2)
C2	14(3)	14(3)	16(3)	3(3)	2(2)	8(3)

Table S9. Statistics of SHG Intensity and TG of Cd-Based 3D Metal-Organic Complexes.

Compounds	Space group	SHG (×KDP)	TG (°C)	Ref.
$(Me_2NH_2)[CdLi(m\text{-}BDC)_2]$	$Pm2$	4.0	116	10
$Cd(3,3\text{–}AZDB)_2\cdot(H_2NMe_2)(NH_4)$	$C222_1$	4.5	220	11
$[Cd2(2,2',3,3'\text{–}ODPA)(bpy)(H_2O)_3]\cdot(H_2O)_2$	$P2_1$	2.4	250	12
$(Me_2NH_2)[CdLi(OBA)_2]$	$I2d$	5.0	264	10
$Cd_2(DPA)_2(mbix)$	$P6_1$	2.1	300	13
$\{[Cd_5(bta)_4]\cdot6H_2NMe_2\}_n$	$I3d$	0.8	300	14
$Cd[(S,S)\text{-}C_{14}H_{14}N_2O_6]$ [Cd(S)]	$C222_1$	9.0	340	15
$Cd[(R,R)\text{-}C_{14}H_{14}N_2O_6]$ [Cd(R)]	$C222_1$	8.0	340	15
$[CdCa(m\text{-}BDC)_2(DMF)_2]$	$Pna2_1$	1.5	345	16
$[Cd(HL)]_n$	$Pna2_1$	0.3	380	17
Cd(AmTAZ)Cl	$P2_12_12_1$	0.1	380	18
$Cd_2(TPOM)(d\text{-}Cam)_2(H_2O)_2$	$P2_12_12$	2.7	407	19
**Cd(imc)**	$\boldsymbol{Pna2_1}$	**10.0**	**470**	**This work**

Table S10. Calculated hyperpolarizability, distortion index and dipole moment of compounds Cd(imc).

Compounds	Group	Hyperpolarizability (lβ_{max}l)	Distortion index ($\mathcal{D}$)	Dipole moment (μ)
	$[CdO_3N_2]$	630.112	4.402	3.313
**Cd(imc)**	$[C_4H_2N_2O_2](imc^{2-})$	2192.536	\	\
	total	3323.884	\	\

Table S11. The SHG contribution of the compound Cd(imc) after RSAC treatment.

Compounds	Group	d_{15}	d_{24}	d_{33}
	$[CdO_3N_2]$	1.143 (23.97%)	-2.118 (38.66%)	1.179 (8.88%)
**Cd(imc)**	$[C_4H_2N_2O_2](imc^{2-})$	3.627 (76.03%)	-3.360 (61.34%)	12.106 (91.12%)
	total	4.199	-3.991	11.189

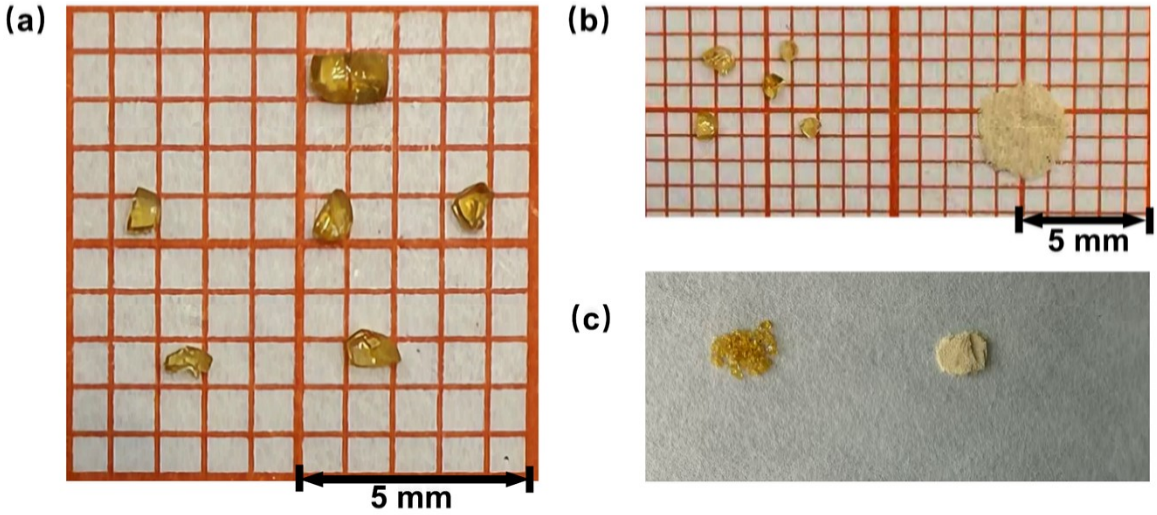

Fig. S1. Color comparison of crystals and powder. (a) Bulk crystals; (b) color comparison of bulk crystals (left) and crystal powder (right) under the same light source; (c) color comparison of bulk crystals (left) and crystal powder (right) under natural light.

As shown in Fig. S1a, the bulk single crystals exhibit a yellow appearance under the photography light source. When ground into powder under the same lighting conditions (Fig. S1b), the color is notably weakened, with the powder appearing light beige. Under neutral natural light (Fig. S1c), the yellow hue of the powder is further diminished.

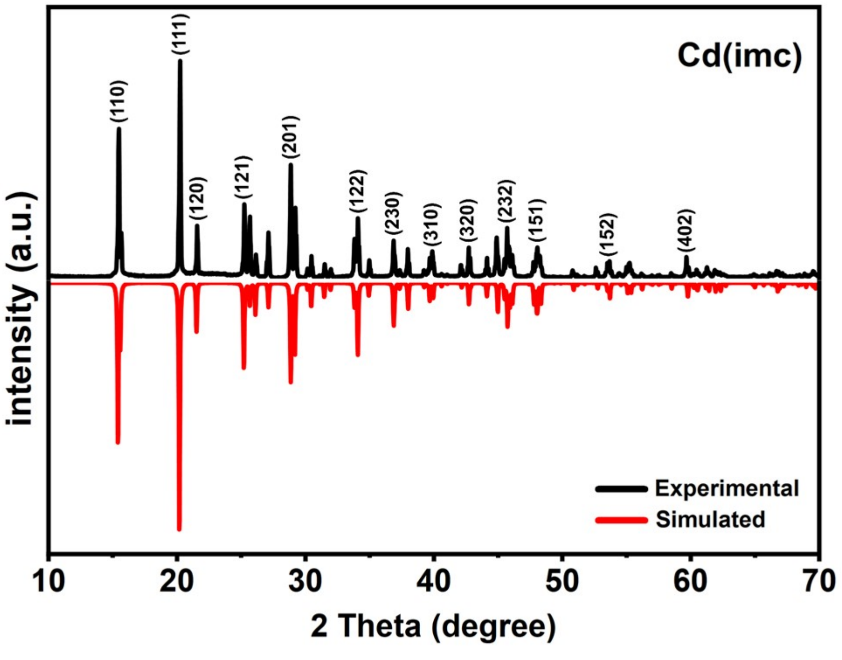

Fig. S2. Experimental and calculated powder XRD patterns of Cd(imc).

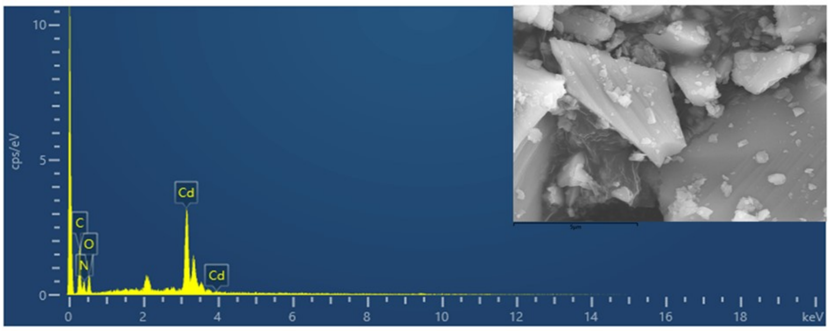

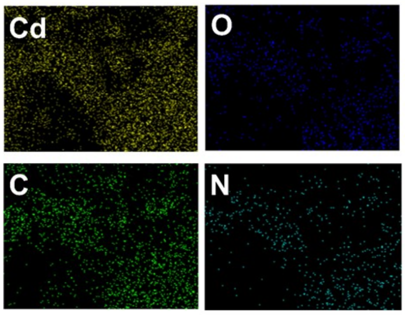

Element	wt%	Wt% Sigma	At%
Cd	55.2	1.2	12.9
C	21.3	0.9	46.5
O	14.5	0.9	23.8
N	9.0	1.1	16.8
total	100.0		100.0

Fig. S3. Scanning electron microscope image of Cd(imc) and its elemental distribution.

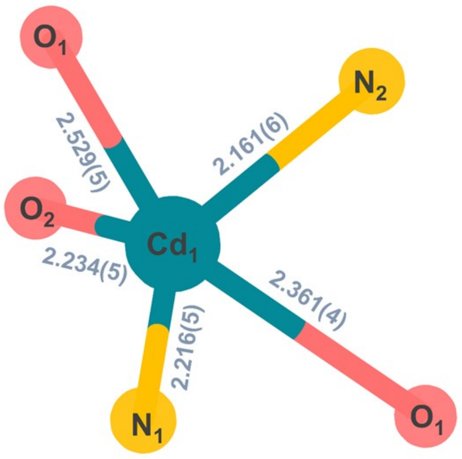

Fig. S4. Coordination environment of Cd^{2+} in Cd(imc).

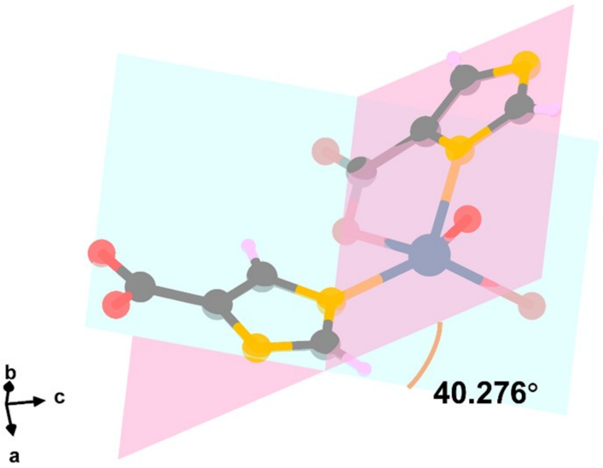

Fig. S5. The dihedral angle between the organic rings in Cd(imc).

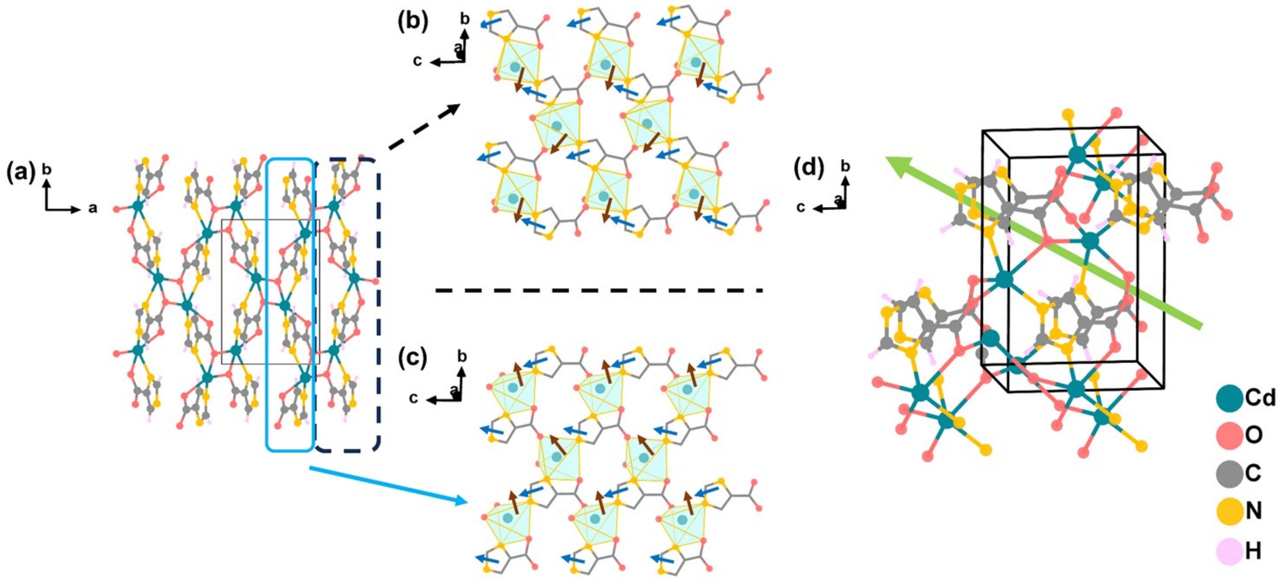

Fig. S6. Dipole moment orientations in different planes of Cd(imc). (a) Crystal structure in the *ab* plane viewed along the crystallographic *c*-axis; (b) Dipole moment orientations of the organic ligands and Cd coordination polyhedra in the plane indicated by the black box in (a); (c) Dipole moment orientations of the organic ligands and Cd coordination polyhedra in the plane indicated by the blue box in (a). (Blue arrows correspond to the dipole moments of the imc^{2-} ligands, and brown arrows denote those of the Cd coordination polyhedra). Schematic diagram of net dipole moment orientation of Cd(imc) (Green arrows represent dipole moment directions).

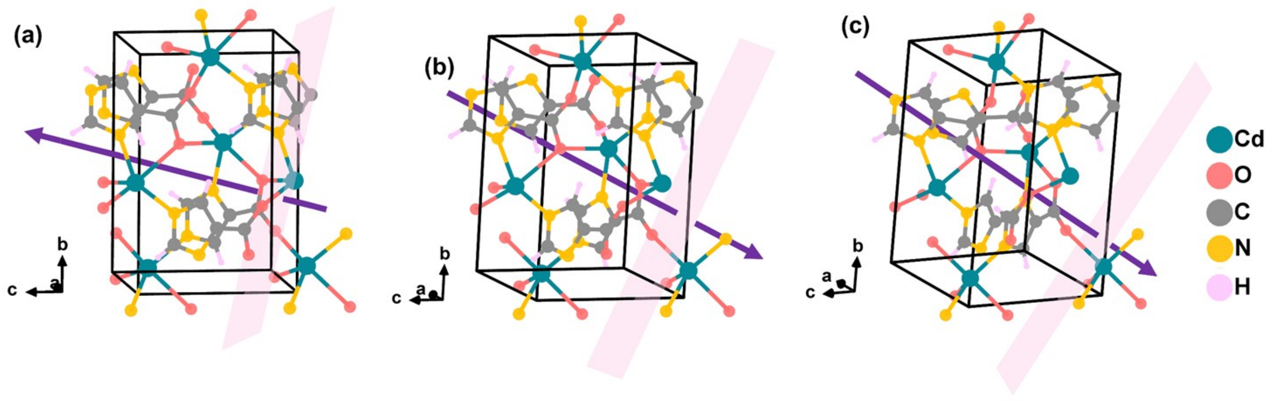

Fig. S7. Directions of the non-zero SHG tensor components in Cd(imc). (a) Direction of the d_{15} SHG tensor component [113]; (b) Direction of the d_{24} SHG tensor component [223]; (c) Direction of the d_{33} SHG tensor component [333]. (The pink plane indicates the effective plane corresponding to each tensor component, and the purple arrow denotes the corresponding SHG polarization direction).

Fig. S6b and S6c, the dipole moment orientations of the Cd coordination polyhedra and imc^{2-} ligands have been added. It can be clearly seen that the dipoles of the two layers form a certain angle, resulting in partial cancellation. However, the direction where the cancellation occurs is not the effective direction of the SHG tensor components d_{15}, d_{24}, and d_{33}, and thus does not weaken the SHG response of the crystal.
Fig. S7 details the effective direction of each non-zero SHG tensor component in Cd(imc), further confirming that the partial dipole cancellation in the two layers with approximate antiparallel arrangement in Fig. S6 has negligible impact on the SHG contribution.
The schematic diagram of the net dipole moment orientation of the Cd(imc) complex in Fig. S6d again demonstrates that, despite the local non-ideal arrangement, the Cd(imc) complex still exhibits a well-defined macroscopic net polarity direction (green arrow).

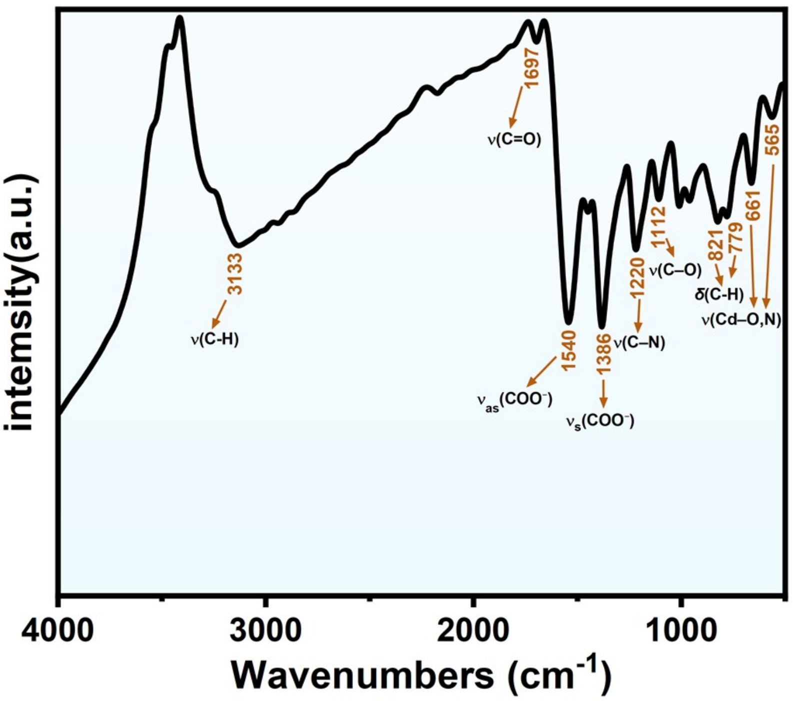

Fig. S8. IR spectra of Cd(imc).

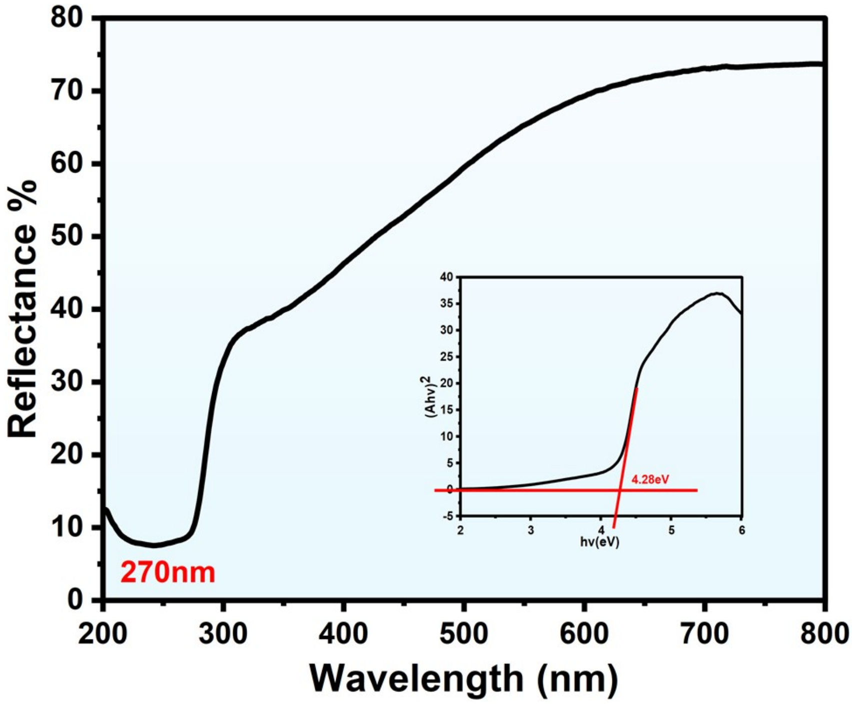

Fig. S9. UV-Vis diffuse reflectance spectra of Cd(imc).

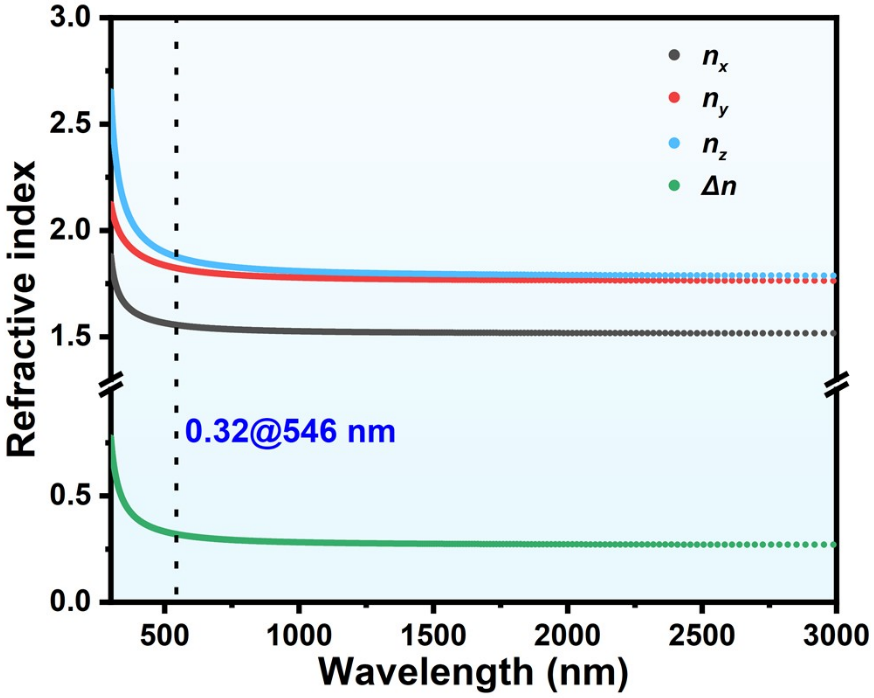

Fig. S10. Theoretically calculated birefringence of Cd(imc).

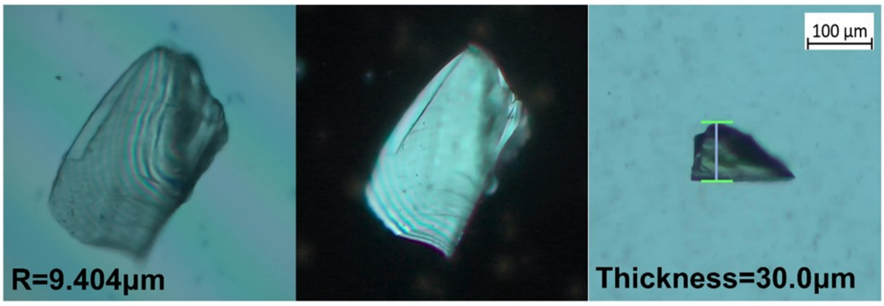

Fig. S11. Photographs of the tested Cd(imc) crystals for birefringence. (from left to right are photos of completely matted, original crystals, thickness).

References

1 V. Dolomanov, L. J. Bourhis, R. J. Gildea, J. A. K. Howard and H. Puschmann, *J. Appl. Crystallogr.*, 2009, **42**, 339–341.
2 G. M. Sheldrick, *Acta Crystallogr., Sect. C: Struct. Chem.*, 2015, **71**, 3–8.
3 M. D. Segall, P. J. D. Lindan, M. J. Probert, C. J. Pickard, P. J. Hasnip, S. J. Clark and M. C. Payne, *J. Phys.: Condens. Matter*, 2002, **14**, 2717–2744.
4 J. P. Perdew, K. Burke and M. Ernzerhof, *Phys. Rev. Lett.*, 1996, **77**, 3865–3868.
5 D. J. Chadi, *Phys. Rev. B*, 1977, **16**, 1746–1747.
6 M. Caricato, E. Frisch, J. Hiscocks and M. J. Frisch, *Gaussian 09: IOps Reference*, Gaussian lnc., Wallingford, CT, 2009.
7 S. Chiodo, N. Russo and E. Sicilia, *J. Chem. Phys.*, 2006, **125**, 104107.
8 Z. S. Lin, X. X. Jiang, L. Kang, P. F. Gong, S. Y. Luo and M. H. Lee, *J. Phys. D: Appl. Phys.*, 2014, **47**, 253001.
9 M. H. Lee, C. H. Yang and J. H. Jan, *Phys. Rev. B*, 2004, **70**, 235110.
10 J. D. Lin, X. F. Long, P. Lin and S. W. Du, *Cryst. Growth Des.*, 2010, **10**, 146–157.
11 Z. F. Chen, R. G. Xiong, B. F. Abrahams, X. Z. You and C. M. Che, *Dalton Trans.*, 2001, **17**, 2453–2455.
12 S. Q. Zang, Y. Su, Y. Z. Li, Z. P. Ni and Q. J. Meng, *Inorg. Chem.*, 2006, **45**, 174–180.
13 G. X. Liu, K. Zhu, H. M. Xu, S. Nishihara, R. Y. Huang and X. M. Ren, *CrystEngComm*, 2009, **11**, 2784–2796.
14 L. Cheng, H. Y. Hu, L. M. Zhang and S. H. Gou, *Inorg. Chem. Commun.*, 2012, **15**, 202–207.
15 J. Kee and K. M. Ok, *Angew. Chem. Int. Ed.*, 2021, **60**, 20656–20660.
16 J. D. Lin, S. T. Wu, Z. H. Li and S. W. Du, *Dalton Trans.*, 2010, **39**, 10719–10728.
17 L. H. Wang, L. Z. Liu, Z. Z. Yao, Y. X. Ye, X. L. Ma, Q. H. Chen, Z. J. Zhang and S. C. Xiang, *Inorg. Chem. Commun.*, 2017, **80**, 49–52.
18 W. Li, H. P. Jia, Z. F. Ju and J. Zhang, *Cryst. Growth Des.*, 2006, **6**, 2136–2140.
